# DNA Methylation Validation Methods: a Coherent Review with Practical Comparison

**DOI:** 10.1186/s12575-019-0107-z

**Published:** 2019-10-01

**Authors:** Šárka Šestáková, Cyril Šálek, Hana Remešová

**Affiliations:** 1 Institute of Clinical and Experimental Hematology, First Faculty of Medicine, Charles University and Institute of Hematology and Blood Transfusion, Prague, Czech Republic; 2 Institute of Hematology and Blood Transfusion, Prague, Czech Republic

**Keywords:** DNA methylation, Validation methods, MSRE, Pyrosequencing, MS-HRM, qMSP

## Abstract

Here, we present a practical overview of four commonly used validation methods for DNA methylation assessment: methylation specific restriction endonucleases (MSRE) analysis, pyrosequencing, methylation specific high-resolution DNA melting (MS-HRM) and quantitative methylation specific polymerase chain reaction (qMSP). Using these methods, we measured DNA methylation levels of three loci in human genome among which one was highly methylated, one intermediately methylated and one unmethylated. We compared the methods in terms of primer design demands, methods’ feasibility, accuracy, time and money consumption, and usability for clinical diagnostics.

Pyrosequencing and MS-HRM proved to be the most convenient methods. Using pyrosequencing, it is possible to analyze every CpG in a chosen region. The price of the instrument may represent the main limitation of this methodology. MS-HRM is a simple PCR-based method. The measurement was quick, cheap and very accurate. MSRE analysis is based on a methylation specific digestion of DNA. It does not require a bisulfite conversion of DNA as the other methods. MSRE analysis was very easy to perform, however, it was not suitable for intermediately methylated regions and it was also quite expensive. qMSP is a qPCR-based method that uses primers designed specifically for methylated and unmethylated alleles of a chosen region. This was the least accurate method and also the primer design and optimization of PCR conditions were highly demanding.

**Electronic supplementary material:**

The online version of this article (10.1186/s12575-019-0107-z) contains supplementary material, which is available to authorized users.

## Background

DNA methylation plays a fundamental role in many crucial biological processes such as embryonic development, gene imprinting, and gene expression regulation. In mammals, the DNA methylation occurs almost exclusively in CpG dinucleotides where a methyl group is attached to the fifth carbon of cytosine base, creating 5-methylcytosine. The biological effect of DNA methylation depends not only on its presence or absence but mainly on its exact location in the genome [1]. Aberrant DNA methylation has been proved as an inducing mechanism in many cancers and is connected to other complex disorders (e.g. diabetes and cardiovascular diseases, neurodegenerative and psychiatric disorders) [2]. Therefore, DNA methylation profiles are examined as biomarkers for diagnosis, prognosis, treatment response and disease monitoring [3, 4].

Nowadays, there is a rapid expansion of high-throughput methods for DNA methylation assessment with single-base resolution. Array techniques can examine as much as 850,000 CpGs at once [5] and all CpG sites, over 28 million in human genome [6], can be analyzed using whole-genome bisulfite sequencing. These methods provide not only an overview of the methylation status of a certain genome, but also a methylation level of each studied CpG. Despite the advantages of these genome-wide approaches, it is still essential to have a proper technique for validation of DNA methylation results for chosen loci. The ideal validation method should be sensitive, quick, cost effective and suitable for screening of large sets of clinical samples to acquire statistically significant data.

In this review, we assessed the methylation status of certain CpGs using four most common methods for DNA methylation validation. These methods were: quantitative PCR with prior digestion by methylation specific restriction endonucleases (MSRE), pyrosequencing, methylation specific high-resolution DNA melting (MS-HRM) analysis, and quantitative methylation specific polymerase chain reaction (qMSP). For proper evaluation of each method, we selected 3 distinct CpG sites within the human genome that were > 99% methylated (methylated “M” locus), around 50% methylated (intermediately methylated “IM” locus), and < 1% methylated (unmethylated “U” locus).

## Overview of Evaluated Validation Methods

### Methods Based on DNA Digestion by MSRE

Restriction methods for quantification of DNA methylation are simple, rapid and do not require bisulfite conversion of DNA. Selective digestion of DNA by methylation specific restriction enzymes (HpaII, AatII, ClaI, etc.) was historically the first method used for assessing DNA methylation levels [7]. High specificity is characteristic for this method, however, only the specific restriction sites can be analyzed which is an important limitation.

The analysis is based on a selective DNA cleavage by MSRE which will not cut its restriction site when a methylated cytosine is present. The most frequently used enzyme is HpaII with recognition sequence CCGG. It is also possible to use a pair of isoschisomeric enzymes, where one is methylation sensitive and the other is not. Most common pair is HpaII/MspI where MspI also cleaves CCGG sequence but regardless of its methylation status. In older protocols, resulting fragments were analyzed on a gel or by a southern blot and the location of methylated sites was estimated from the fragments’ sizes [8, 9]. Newer approaches employ quantitative PCR (qPCR) [10]. In order to determine methylation of a specific region, DNA is digested by MSRE and subsequently analyzed with qPCR using primers surrounding the sequence of interest. Methylation percentage is counted from threshold cycles (C_t_) measured for digested and undigested control DNA. For this approach, it is possible to buy easy-to-use commercial kits with mixes of MSREs to target more sites, e.g. *OneStep* qMethyl kit from Zymo Research [11].

Primers can be easily designed with free online software such as Primer3Plus [12] (*http://www.bioinformatics.nl/cgi-bin/primer3plus/primer3plus.cgi*) or Primer-BLAST [13] (*https://www.ncbi.nlm.nih.gov/tools/primer-blast/*). However, it is required that at least two restriction sites are inside the amplicon to reliably measure the DNA methylation. Thus, it is not possible to investigate the methylation level of only one particular CpG site.

### Methods Requiring Bisulfite Converted DNA

Bisulfite (BS) conversion of DNA is a crucial step in most DNA methylation analyses. Already in 1970, it was discovered that sodium bisulfite mediates the deamination of cytosine into uracil while the methylated cytosine is left intact [14]. After PCR amplification, the converted residues are read as thymines and methylated cytosines will remain cytosines. It is important to note that after BS conversion the DNA strands are no longer complementary. This must be kept in mind when choosing a method for quantification of BS converted DNA.

The reliability of methylation analysis is dependent on a complete BS conversion. Unconverted cytosines, if present, would be mistaken for methylated loci and the analysis will produce biased results. Formerly, the conversion method required a high DNA input and exposure to high bisulfite salt concentration under high temperatures and low pH. These harsh conditions resulted in significant DNA fragmentation and loss [15]. Nowadays, there is a wide variety of commercial kits available that are able to convert as low as 100 pg of DNA in less than 2 h. These kits, nice comparison is available here [16], use convenient column system and guarantee more than 99% conversion efficiency.

#### Pyrosequencing

Pyrosequencing is a sequencing method used for quantitative methylation analysis of bisulfite converted DNA. For its relative simplicity, speed and comparable results, pyrosequencing can be preferred to cloning [17], a method used as a gold standard for identification of allele specific methylation patterns [18]. Another advantage of pyrosequencing is that it is suitable for both CpG poor and CpG rich regions. Main drawback of this method is that only shorter regions (maximum 350 bp) can be analyzed. However, this disadvantage can be overcome by using more sequencing primers on one amplicon or by a serial pyrosequencing [19, 20].

Pyrosequencing process can be divided into three steps: (i) PCR amplification and tagging using a biotinylated primer, (ii) isolation of the PCR product with streptavidin beads and hybridization with a sequencing primer, and (iii) sequencing. During the sequencing step, nucleotides are added in a predefined order depending on the sequence of interest. The technology is based on a release of pyrophosphate (PPi) during nucleotide incorporation when complementary to the template DNA strand (the purified PCR product). An ATP sulfurylase then uses PPi and adenosine phosphosulfate to produce ATP. ATP is utilized by luciferase which converts luciferin to oxyluciferin. The intensity of produced light is detected and translated as a peak on a pyrogram [21]. Methylation percentage is then calculated from the ratio of heights of a cytosine peak (methylated signal) and the sum of cytosine and thymine peaks (methylated and unmethylated signal) for each cytosine in a CpG dinucleotide.

As mentioned above, this method is suitable for regions 80–200 bp long. One reason is that longer amplicons could form secondary structures and loops that would impede the sequencing reaction. The second issue arises during the sequencing procedure where nucleotides are added in each sequencing cycle. The volume in reaction wells increases which causes dilution of all reagents and thus a decrease of the signal. At the same time, the background signal rises during the sequencing due to an incomplete degradation of previously added nucleotides [21]. Because of that, a signal measured after 90–100 cycles has low quality and the results are not credible [20].

Having a strong amplicon with no side product, and therefore a high-quality primer design, is crucial for this assay. One way is to order the primers from commercial companies. For example, Qiagen offers a full assay design for desired regions and it is also possible to buy a predesigned primer set. The other option is to use a free software for bisulfite primer design such as MethPrimer [22] (*http://www.ucsf.edu/urogene/methprimer/index1.html*), Bisearch [23] (*http://bisearch.enzim.hu/*) or MethylPrimer Express by Applied biosystems (*http://www.appliedbiosystems.com/methylprimerexpress*). Moreover, it is important to check for potential primer dimers formation or self-complementarity of the primers, e.g. with Multiple Primer Analyzer (*https://www.thermofisher.com/cz/en/home/brands/thermo-scientific/molecular-biology/molecular-biology-learning-center/molecular-biology-resource-library/thermo-scientific-web-tools/multiple-primer-analyzer.html*). Primers should be 15–30 bp long (20 bp is optimal) with a melting temperature between 50 and 69 °C (optimally 60 °C) [24]. There should be at least four non-CpG cytosines in each primer to assure that only a properly BS converted DNA will be amplified. Presence of a CpG and therefore a use of a degenerated primer in not recommended because it may lead to a preferential amplification of a specific subset of molecules [21]. However, in some of our previous experiments we used degenerated primers without any difficulties. One of the PCR primers must be labeled on its 5’end by biotin and this primer should be purified by HPLC or an equivalent procedure to assure zero contamination by free biotin molecules. The orientation of a sequencing assay depends on which primer is tagged. It is also essential to incorporate all biotinylated primers into amplicons during the PCR step. Otherwise, these primers might compete with the amplicons during the streptavidin binding. It is recommended to use 0.1 μM biotinylated primer and 0.2 μM unlabeled primer concentrations and 45–50 PCR cycles. It is also possible to use a universal biotinylated primer and a tailed reverse primer in 5:(0.01–1) ratio [20, 25]. The use of a universal biotinylated primer significantly reduces the costs when having more pyrosequencing assays for various regions. On the other hand, it sometimes requires deeper PCR optimization to gain a sufficient amount of the PCR product. The amount and size of the amplified PCR product as well as a negative PCR control should be always checked by an agarose gel electrophoresis to prevent further complications.

Sequencing primer should be 15–20 bp long with a melting temperature between 45 and 55 °C. The most relevant part of the primer are the last four or five bases on the 3’end which should be unique in the amplicon. Also, it is not recommended to use the non-biotinylated PCR primer as a sequencing primer. The sequencing primer should differ from the PCR primer in at least one additional nucleotide on the 3’end [21]. Nevertheless, we tried to use the non-biotinylated primer as a sequencing primer in some of our previous experiments and the pyrosequencing was successful.

A set of assay validation reactions, listed in appendix B of PyroMark Q24 User Manual 2016, should be always performed before using the assay to analyze samples. These controls are (i) PCR reaction without template DNA, (ii) PCR product without sequencing primer, (iii) sequencing primer without PCR product, (iv) biotinylated primer without PCR product, and (v) sequencing primer and biotinylated primer together without PCR product. Moreover, in each assay, controls of BS conversion should be included in the dispensation order [21]. The BS conversion ratio can be evaluated when a dispensation of cytosine nucleotides is incorporated before or after thymines which are supposedly converted cytosines in the sequence. In case of an unsuccessful BS conversion, peaks would appear in the pyrogram for these additional dispensations.

#### Methylation Specific HRM Analysis

MS-HRM is a method based on different melting temperatures (T_m_) of methylated and unmethylated DNA. T_m_ is defined as a temperature at which the two DNA strands dissociate and is characterized by a sudden drop of fluorescence signal due to a release of an intercalating dye, e.g. SYBR Green, EvaGreen or SYTO9. T_m_ depends on the DNA base composition because CG base pairs are connected by three hydrogen bonds and AT pairs only by two. This also enables to differentiate between methylated and unmethylated DNA after a BS conversion where the unmethylated cytosines are converted to uraciles and after PCR changed to thymines.

MS-HRM comprises of PCR for amplification of a chosen region followed by HRM analysis with ramping by only 0.1 °C [26]. It is recommended to use quantitative PCR for the amplification as an additional quality control [27]. For the DNA methylation assessment, DNA standards are analyzed together with the samples. Standards are prepared by diluting fully methylated BS converted DNA by fully unmethylated BS converted DNA and are usually 100, 75, 50, 25, 10 and 0% methylated. By comparing the HRM curves of standards and samples it is possible to determine an approximate methylation level [26]. Several more quantitative approaches for establishing the DNA methylation were developed. It is possible to construct a linear curve by plotting the temperature at which 50% of DNA is dissociated (T_50_) against the methylation percentage of the standards [28]. Another method estimates the methylation level by using two sets of primers, methylated and unmethylated, for amplification. A differential melting profile is then calculated by normalizing the methylated HRM profile against the unmethylated. The differential fluorescence peak heights are then plotted against the dilution factor which generates a linear calibration curve [29]. Another approach uses peak heights and area under the curve (AUC) of normalized and temperature shifted first derivatives of HRM curves. There is a linear dependency between these values and the methylation percentage [27].

For MS-HRM analysis, the only requirement are primers surrounding the region with CpGs of interest. It is again crucial to obtain a pure PCR product. Primers should be between 15 and 30 bp long with similar melting temperatures around 65 °C. This will allow to run the PCR at approximately 60 °C which is important for the method’s specificity [30]. Generally, for this type of primers that surround the region of interest, it is not advised to have a CpG inside the primer. However, Wojdacz et al. have shown that inclusion of a CpG in the primer sequence can compensate the PCR bias towards unmethylated alleles and thus significantly increase the method’s sensitivity [30, 31]. They also claim that MS-HRM is taken as a method to assess methylation levels and therefore a slight bias towards the methylated sequences further increases the method’s sensitivity [32]. According to their recommendations, the primers should contain one or two CpG dinucleotides as close to their 5’end as possible [33]. It is necessary to have several thymines, corresponding to unmethylated cytosines, included in the primer sequence to amplify only properly converted DNA. It is also advisable to check for primer dimers and loops formation. The amplicon should be kept small, around 100 bp, to reduce the complexity of its melting profile [32]. Nevertheless, it should be considered that a shorter PCR product gives higher sensitivity but limited resolution between methylation levels because of only small difference between methylated and unmethylated DNA. Longer amplicons have more distinguishable methylation profiles [26].

#### Methylation Specific PCR

In this methodology, DNA methylation is examined by two sets of primers where one is specific for a methylated state (Met) and the other pair for an unmethylated state (Unm) of a certain genomic locus. A set of two PCR reactions is performed and the products are analyzed via a gel electrophoresis [34].

Despite the relative simplicity of this method, finding convenient methylated and unmethylated primers is sometimes challenging. The primers are designed to span the analyzed region and should contain from one to three CpGs ideally located at the 3’end of the primer. Therefore, this method is more suitable for CpG rich regions, like CpG islands. There should be again at least five thymines, BS converted unmethylated cytosines, included in the sequence to assure that only a properly BS converted DNA will be amplified [16]. Primers length should be at least 23 bp with melting temperature between 55 and 65 °C. It is recommended that Met and Unm primers have similar melting temperatures. To achieve that, it is often needed to prolong the Unm primer on the 5’end because the BS conversion decreases the T_m_ of unmethylated DNA [35]. The above-mentioned software for finding bisulfite primers for pyrosequencing or MS-HRM can be also used for designing MSP primer sets.

During the PCR, the number of amplification cycles should not exceed 35 because after that a false methylation signal could appear. It is also crucial to use such annealing temperature (T_ann_) that the primers are specific for the DNA methylation status they were designed for [35]. Thus, it is essential to perform proper control reactions in each new MSP experiment such as PCR with both methylated and unmethylated standards, no template control and PCR with non-converted DNA [16].

For a long time, this method was only qualitative. As a result of the electrophoresis, it was possible to recognize that only methylated or unmethylated locus or both loci were amplified. Previous studies did not find any clear correlation between the size of the band on the gel and the amount of DNA examined. Nevertheless, this does not compromise the great sensitivity of this method [16]. Newer protocols employ quantitative PCR and make this method semiquantitative or quantitative. For example, for establishing unmethylated DNA, qPCR is performed with Unm primers together with bisulfite specific primers (BSP) that amplify chosen locus independently of its methylation status. The ratio of unmethylated alleles to total number of amplified molecules is then calculated by either classical ΔΔC_t_ approach with or without correction for primers efficiency [36] or by a demethylation index, as proposed in another study [37]. Apart from using simple qPCR with an intercalating dye, a quantitative method called MethylLight was developed. It uses a pair of methylation specific TaqMan probes where each probe, designed for either methylated or unmethylated DNA, is tagged with different fluorophore [38]. When using quantitative MSP approaches, it is advisable to perform a melt analysis after the PCR to check for any side products [35].

## Results and Discussion

### MSRE Analysis

**Fig. 1:**
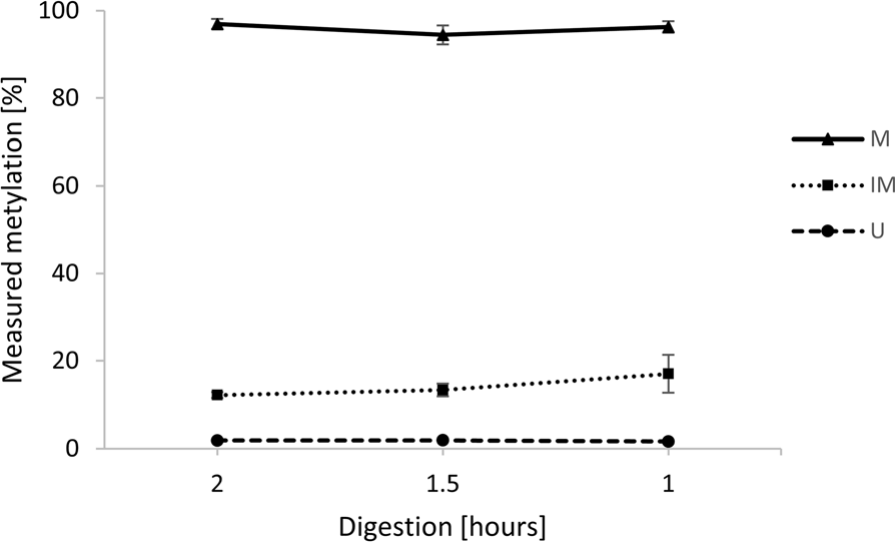
The influence of digestion time on measured methylation levels in MSRE analysis. Error bars represent the standard deviation (*n* = 4). M - methylated locus, IM – intermediately methylated locus, U – unmethylated locus

With the MSRE approach, we were able to accurately measure methylation levels of the M and U loci. However, for the IM locus, we acquired lower methylation levels than expected. Therefore, we performed two additional MSRE experiments with shortened digestion time to see whether it will increase the methods accuracy for the IM locus. The recommended digestion time was 2 h, so we additionally tried 1.5-h and 1-h digestions. There was no statistically significant difference in DNA methylation levels measured after various digestion times. We achieved only a slight improvement for the IM locus, where the calculated methylation percentage rose from 12 to 17% when the digestion time was shortened to 1 h. The methylation levels for M and U loci remained the same. The results for all three loci are shown in Fig. 1. Based on these results, we propose that a shorter digestion time may be used to make the MSRE method faster while retaining the original results.

### Pyrosequencing

In the pyrosequencing procedure, the most important step for a successful analysis is gaining a strong amplicon. However, even when we detected a strong band on our agarose gel after the PCR, we did not achieve the desirable signal during pyrosequencing. Thus, we tried to enhance the binding of the amplified, biotin-labeled PCR product by adding more streptavidin beads into the process. We compared the results after adding 1, 2 and 3 μl of streptavidin beads per sample. The 2 μl proved to be ideal for gaining the strongest signal on the pyrogram. Additionally, we prolonged the agitation step to 20 min to increase the number of bound molecules. Also, we noticed that after the agitation, it is essential to proceed immediately to the next step to ensure that the beads are resuspended in the tube and will be taken up efficiently by the probes in the subsequent procedure. According to the manufacturer, for accurate results the peak height of a single base should be at least 40 units in the pyrogram. From our experience, experiments where single based peaks are at least 25 units high give reliable results. Nevertheless, when a strong amplicon was acquired, judging by the results of the agarose gel electrophoresis, the peak height of a single base was always around 50–200 units.

In the resulting pyrograms, it was obvious that the signal starts dropping significantly after 45th dispensation cycle which roughly corresponds to a 100 bp region. This is in accordance with the recommendations for this method to keep the studied region short [20].

In our experiments, we measured four CpGs in the M locus and all were highly methylated (95.4 ± 3.1%). In the U locus, we measured three CpGs and all were unmethylated (7.4 ± 3.1%). There were only two CpGs in the IM locus. The IM CpG chosen from the Infinium MethylationEPIC BeadChip data was indeed intermediately methylated 58.5 ± 7.3%. However, the next CpG included in the sequenced amplicon was rather unmethylated 18.4 ± 3.8%. The final average methylation of the studied region was thus around 37%. This demonstrates the main advantage of the pyrosequencing method which is the base resolution. The other quite beneficial aspect of pyrosequencing is the bisulfite conversion control which allows us to see whether the BS conversion was done properly [21]. We always included at least three of these BS control dispensations in our pyrosequencing assays.

### MS-HRM

Wojdacz et al. have shown that inclusion of a CpG to the primer sequence can compensate the PCR bias of unmethylated alleles by favoring amplification of methylated alleles [32]. Thus, we designed two sets of HRM primers for the M and U loci. One primer set did not include any CpGs in its sequence. The other set was designed according to Wojdacz et al. [30] and each primer had one or two CpGs on its 5’end. It was not possible to design Wojdacz HRM primers for the IM region because of its CpG shortage. Sequences of the primers are listed in Table 1.

**Table 1:** Primer sequences and characteristics

Primer type	Forward/ sequencing primer	Reverse primer	T_ann_ [°C]	Product length [bp]
M pyrosequencing	GGTAGGAGGATGGTTTGAATT/ GGTGGAAATGAAGTAGGTGTGTTTG	*GTGCCGAGGCTCAGGC*AACACTACTCTTACCAAAACAACC	60	373/ 227
IM pyrosequencing	GTTAAGGGGGTGTATTTTAGAGA/ GGTAGAGAGAAGTTTTTTTTGTAGG	*GTGCCGAGGCTCAGGC*CTTAACTACTTTCCCAAACTACCT	58	399/ 339
U pyrosequencing	GGGGGGGTGTTAGTATTTG/ TTAGTATTTGYGTTGTGGAGTG	*GTGCCGAGGCTCAGGC*CCAAACTAACCTAATAAAACC	58	300/ 290
Universal biotinylated primer	5’biotin-*TCTGTGCCGAGGCTCAGGC*			
M MSRE	TTTTCTGTGACCTCCTTTGG	CAGTGTGACTGCTGGTGAAG	60	243
N MSRE	GCAATAGGCGTTAATGTCGT	AGGAGTGGCAAAAGAGGACT	60	199
U MSRE	CGCTTAGCAATCATCGACTT	GAAACAGGCCGCATCCTC	60	265
M MSP Met	GTATATTCGGAATTATTTCGTTTTC	AATTAACAACCGACAACCG	56	72
M MSP Unm	GATGTATATTTGGAATTATTTTGTTTTT	AATTAACAACCAACAACCA	56	75
IM MSP Met	CGGTTTTTATAGTTTTGAATTAGATC	TTATTTATTATCACATCAACTACTTCCG	58	166
IM MSP Unm	ATTGGTTTTTATAGTTTTGAATTAGATT	TTATTTATTATCACATCAACTACTTCCA	58	168
U MSP Met	CGTTGTGGAGTGAAGTGAATC	ACCGAACGAACAATAAACGAA	54	210
U MSP Unm	TGTGTTGTGGAGTGAAGTGAATT	ACCAAACAAACAATAAACAAAAAA	54	212
M HRM	TTGGGTGGAAATGAAGTAGGTGTG	CCAAACCATTAACCATAACAATA	54–58^*^	94
IM HRM	TTTGGGGAAAAAATATATGGAGTT	CTACTAATAAAACCCTTTACTCCCA	54–58^*^	90
U HRM	TTAGTATTTGYGTTGTGGAGTG	CCRACACTTACTCTTATTAACRATC	54–58^*^	93
M HRM Wojdacz	**CG**GGGGGGTGTTAGTATTTG	CC**CG**ACACTTACTCTTATTAACRATC	55	110
U HRM Wojdacz	T**CG**TGTTTTTTTTTGGGTGGAAATG	**GCG**ACCAAACCATTAACCATAACA	55	104

^*^For MS-HRM experiments T_ann_ was 55 °C, in qMSP experiments T_ann_ of MSP primers was used

*M* methylated locus, *IM* intermediately methylated locus, *U* unmethylated locus, *MSP* methylation specific PCR, *Met* primers for methylated DNA sequence, *Unm* primers for unmethylated DNA sequence, *HRM* high resolution melting analysis, *T*_*ann*_ annealing temperature

From acquired MS-HRM data, we constructed calibration curves as proposed by Tse et al. [27] for every primer set. The correlation coefficients (R^2^) for peak height-based calibration curves together with calculated methylation levels for each locus are summarized in Table 2. The AUC-based calibration curves had slightly lower correlation coefficients. This was probably caused by less exact AUC calculations performed in Excel which we used to keep the data analysis as simple as possible. Still, the AUC-based DNA methylation assessment gave similar results as the peak height approach (see Additional file 1).

**Table 2:** Correlation coefficients for peak height-based MS-HRM calibration curves and counted methylation levels

Locus name	Primer set	R^2^	Methylation [%] (*n* = 10)	± SD
M	M HRM	0.952	93.61	5.28
M	M HRM Wojdacz	0.798	85.49	5.13
IM	IM HRM	0.973	29.20	4.71
U	U HRM	0.868	2.69	1.04
U	U HRM Wojdacz	0.938	0.57	0.81

*M* methylated locus, *IM* intermediately methylated locus, *U* unmethylated locus, *R*^*2*^ square of the correlation coefficient, *SD* standard deviation.

Interestingly, the results in Table 2 show that the Wojdacz’s improvement of primers’ sequence was quite beneficial for the U region. However, it caused a deviation in measurements of the M region resulting in worse R^2^ of the calibration curve and lower calculated methylation levels. It is thus not so straightforwardly beneficial to introduce the CpGs into primers’ sequence. Gaining the optimal results apparently require additional thorough T_a_ optimization to achieve equal amplification of methylated and unmethylated alleles with Wojdacz’s primers [26]. Nevertheless, with all primer sets, the methylation levels of all three loci were measured accurately enough.

### qMSP

We designed both Met and Unm primer sets for each locus to perform the qMSP experiments. Reassuringly, in all investigated samples, the M locus was amplified only by Met primers, the U locus only by Unm primers and the IM locus was amplified by both Met and Unmet primer sets. Regarding the DNA methylated and unmethylated standards, all three loci were always amplified only by the corresponding Met or Unm primer set. The HRM primers were used as BSP, amplifying the region independently of its methylation status. Summary of C_t_ values and measured efficiencies for all primer sets is shown in Table 3. We were not able to measure the efficiency for IM Unm primer set properly. The deviation between duplicates was higher probably because the efficiency of the primer set was low and the amplification of the first dilution began always after 34th cycle. The measurement was thus less reliable despite the fact that the resulting calibration curve had *R*^2^ > 0.99. Therefore, we tried to count the efficiency of MSP primers based on C_t_ values measured for standards and an assumption that BSP primers have 100% efficiency. We used the following equation ${E}_c=100\bullet \frac{C_t^{BSP}}{C_t^{MSP}}$. This counted efficiency corresponded well with the measured efficiency (Table 3) and we used it in the subsequent analysis for the IM Unm primer set.

**Table 3:** qMSP C_t_ values and primers’ efficiencies

Locus and used primer set	Average C_t_ of samples (*n* = 10)				C_t_ of standards		Efficiency		
	MSP	± SD	BSP	± SD	MSP	BSP	MSP measured	MSP counted	BSP measured
M Met	23.18	0.52	22.88	0.27	24.01	22.70	96.47	94.57	83.00
IM Met	34.54	1.23	24.33	0.35	29.23	24.51	81.13	83.85	94.78
IM Unm	32.21	0.50	24.33	0.35	31.99	25.15	125.80	78.63	94.78
U Unm	37.36	0.80	23.04	0.20	37.95	23.40	65.93	61.66	90.52

*M* methylated locus, *IM* intermediately methylated locus, *U* unmethylated locus, *Met* primers for methylated DNA sequence, *Unm* primers for unmethylated DNA sequence, *MSP* methylation specific primers, *BSP* bisulfite specific primers (methylation independent), *SD* standard deviation.

We analyzed our data using all three approaches reviewed by Housseiny et al. [36]. The relative expression ratio method, developed originally by Pfaffl [39], gave very variable results with extremely high standard deviation and thus was not reliable (see Additional file 2). The other approaches, demethylation index and ΔΔC_t_, gave quite similar results, reviewed in Table 4. The M locus was highly amplified by the MSP Met primers. The seemingly double amplification of MSP primers compared to BSP primers indicated by ΔΔC_t_ ≅ 2 is probably caused by the method’s inaccuracy because when we repeated the experiment with five samples, the ΔΔC_t_ results were 1.5 ± 0.3. The ΔΔCt results for U locus were close to 1, meaning that the number of molecules amplified by MSP Unm and BSP primer set was comparable. Regarding the IM locus, the MSP Unm primers amplified around half of the molecules in comparison with the BSP primers, which corresponds with the expected intermediate methylation level of this locus. However, the results of MSP Met primers were spoiled by the disproportionately high C_t_ measured for the methylated DNA standard resulting in a very low ratio of molecules amplified by MSP Met primers in the samples. This could be caused by a higher affinity of Met primers to the methylated DNA standard, compared to samples’ DNA that was rather unmethylated in the IM region.

**Table 4:** Summary of qMSP methylation results calculated using demethylation index and ΔΔC_t_ approach

Locus and used primer set	Demethylation index		ΔΔC_t_	
	Average (*n* = 10)	± SD	Average (*n* = 10)	± SD
M Met	2.02	0.60	2.09	0.66
IM Met	0.05	0.03	0.03	0.02
IM Unm	0.51	0.10	0.50	0.12
U Unm	1.03	0.30	1.30	0.66

*M* methylated locus, *IM* intermediately methylated locus, *U* unmethylated locus, *Met* primers for methylated DNA sequence, *Unm* primers for unmethylated DNA sequence, *SD* standard deviation

### Overall Methods Comparison

**Fig. 2:**
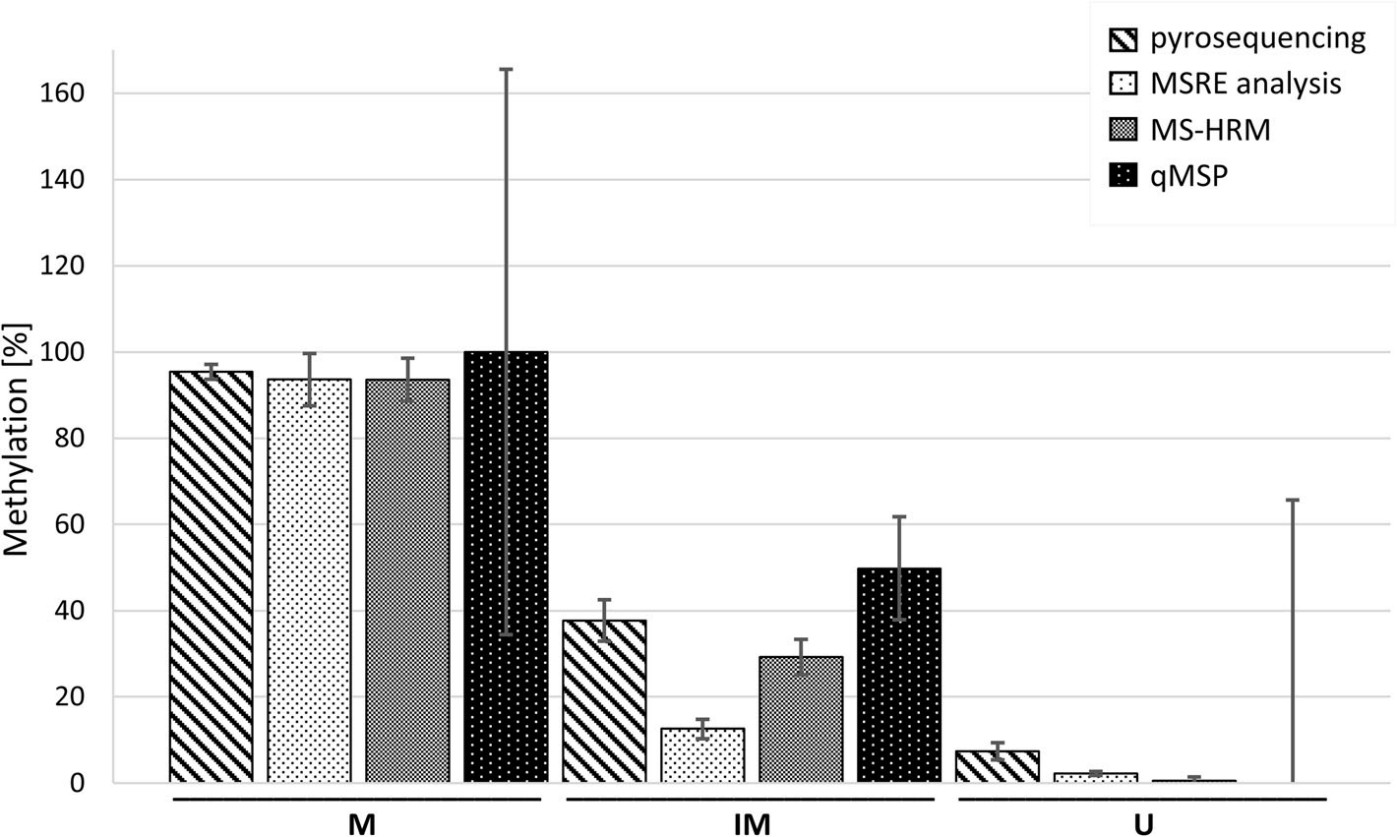
Summary of DNA methylation levels measured by the investigated methods. The average methylation of 10 samples is shown, the error bars represent the SD. Displayed MSRE data were measured after 2-h digestion. Displayed MS-HRM data were acquired using HRM M, HRM IM and HRM U Wojdacz primers. qMSP data shown were calculated using ΔΔC_t_ approach and multiplied by 100 to gain the percentage. For the M locus in qMSP, all values were higher than 100% so we set the mean to 100% to make the figure more comprehensible, the SD was calculated from the original values multiplied by 100. For the IM and U loci in qMSP, we calculated the methylation percentage as 1-(Unm ΔΔC_t_), the SD was also calculated from the original ΔΔC_t_ values multiplied by 100. M – methylated locus, IM – intermediately methylated locus, U – unmethylated locus

The final results of DNA methylation levels measured by all four methods are shown in Fig. 2. All methods were comparable and correlated with each other with *R*^2^ > 0.92 and *p*-value < 1.2·10^− 17^, except the qMSP method results of which were spoiled by extreme standard deviations. We also provide a review of all costs and measurements for each method in Table 5 and a final evaluation of few other parameters in Table 6.

**Table 5:** Costs summary of each method

Method	Total cost of analysis [$]	Number of samples measured	Number of standards measured	Total number of measurements	Cost per measurement [$]
MSRE analysis	576	10 for each locus^*^, Test and Reference reaction, duplicates	2 for each locus^*^, duplicates	144	4
pyrosequencing	162^‡^	10 for each locus^*^	2 for each locus^*^	36	4.5
MS-HRM	85	10 for each locus^*^, duplicates	6 for each locus^*^, duplicates	96	0.9
qMSP	196	10 for each primer set^†^, duplicates	2 for each primer set^†^, duplicates	216	0.9

^*^Number of loci = 3

^†^Number of MSP/HRM primer sets for each locus = 3

^‡^price of the pyrosequencing instrument ca 45,000 $

**Table 6:** Overall evaluation of tested methods

Method	Base resolution	Consistency across methylation levels	Analysis of acquired data	Method optimization	Time consumption	Price
MSRE analysis	–	*	*	*	*	***
Pyrosequencing	+	***	*	*	***	**
MS-HRM	–	***	**	*/**(if needed)	*	*
qMSP	–	**	***	***	**	**

* - simple/low, ** - intermediate, *** - demanding/high

Indisputably, pyrosequencing has most advantages in terms of the DNA methylation assessment of a specific locus. Primer design and interpretation of the results is straightforward with available software. Only the PCR step may require some optimization for gaining a sufficient amplicon but this is not always necessary. A disadvantage of this method may be the relatively high price of the instrument. Also, the method is more time consuming because it comprises three steps: PCR, gel electrophoresis and sequencing itself. This also corresponds with the higher price per one measurement.

When the pyrosequencing instrumentation is unavailable, we recommend using MS-HRM. The primer design is feasible for most regions. In our experiment, we had a CpG poor region (IM) as well as CpG dense regions (M and U) and were able to design reliable sets of primers for both. As we discussed in the chapter about the results from MS-HRM, deep optimization of primers’ sequence and T_ann_ is not needed for method resolution of 5–10%. This method is very simple as well as cost and time effective. The approximate results can be derived immediately from the melting curves. The exact quantification is not so straightforward when a specific software is not provided. Nevertheless, the calculations can be done using free software and Excel, as we have shown.

The MSRE analysis proved to be a quick and simple method. The main advantage of this approach is that it does not require the BS conversion of DNA. Thus, less DNA is needed to perform the analysis and it also makes the primer design easier. We were able to accurately measured the DNA methylation in M and U regions. Obviously though, the method is not suitable for intermediately methylated loci. Even by shortening the digestion time to half of that recommended, the measured DNA methylation of IM locus remained significantly lower than expected. Also, the method is very costly when compared to the other three.

The last method evaluated was qMSP and this caused the most difficulties. The primer design was quite challenging and nearly impossible for the IM locus because of its lack of CpGs. Another issue was to find a suitable T_ann_ at which both Met and Unm primer sets were specific only for the methylated or unmethylated allele respectively but still functional so that it amplified the corresponding DNA standard. When we finally achieved this, the primers had very low efficiency, except for the M Met primer set. Moreover, the exact quantification of measured data was difficult and the results of the M and U loci had extremely high standard deviations within the samples. This method, despite of its simplicity, is also quite expensive because it requires amplification of a chosen region by at least one MSP primer set and BSP primers.

## Conclusion

Even in the era of arrays and next-generation sequencing, it is essential to have a method for validation of acquired DNA methylation data. A quick, cost-effective, and reliable method that would enable to confirm or reject a potential clinical significance of certain methylation changes and could be used in common laboratory practice is still needed.

We tested four standard methods that are used for DNA methylation validation: MSRE analysis, pyrosequencing, MS-HRM and qMSP. In terms of overall feasibility, obtained DNA methylation information and consistency across various methylation levels, we consider pyrosequencing and MS-HRM approaches as the most suitable. Pyrosequencing enables base resolution and thus acquisition of a methylation level for each CpG in the region, an indisputable benefit. MS-HRM can be also designed to investigate a single CpG locus when needed. Otherwise, it provides an overall DNA methylation status of all CpGs inside the studied region. Apparently, MSRE and qMSP are not very applicable for the detection of intermediate levels of DNA methylation. Nonetheless, MSRE does not require BS conversion of DNA and as we showed here, the digestion time can be shortened to one half. This makes the MSRE analysis the simplest and fastest out of the four methods compared. The qMSP approach proved to be quite imprecise and demanding so it may be more convenient to keep this method only as a qualitative tool.

## Materials and Methods

### Characterization of Analyzed CpGs

The three analyzed CpGs with different levels of methylation were selected based on healthy donors’ data from Infinium MethylationEPIC BeadChip (Illumina, San Diego, CA, USA) acquired in our previous work [40]. Characteristic of chosen loci is summarized in Table 7. These CpG dinucleotides were also chosen so that they are within CCGG sequence to enable their cutting by MSRE.

**Table 7:** Specifications of selected CpG sites

Locus name	BeadChip probe ID	Cytosine location (hg 19)		Beta value for all samples measured with BeadChip
		Chromosome	Position	
M	cg24337108	10	11,797,422	> 0.99
IM	cg25722983	1	36,840,028	from 0.45 to 0.55
U	cg09655782	4	57,333,859	< 0.1

*M* methylated locus, *IM* intermediately methylated locus, *U* unmethylated locus, *BeadChip* Infinium MethylationEPIC BeadChip (Illumina), *Beta value* corresponds to methylation percentage

### Samples and DNA Standards

This study was approved by the Institutional Ethics Committee and all blood donors provided their full consent. Mononuclear cells of ten healthy blood donors were harvested from buffy coats by Ficoll gradient centrifugation (Histopaque, Sigma-Aldrich, St.Louis, MO, USA). DNA was extracted using MagCore system (RBCBioscience, New Taipei City, Tchaj-wan). Human Methylated & Non-methylated DNA Set (Zymo Research, Irvine, CA, USA) was used as methylated and unmethylated standards.

### MSRE Analysis

*OneStep* qMethyl Kit (Zymo Research) was used for MSRE analysis. For each sample, DNA (20 ng) was processed through the Test and Reference reactions according to the manufacturer’s protocol. In the PCR step, T_ann_ was set to 60 °C and annealing time was shortened to 45 s. Rotor-Gene Q 2plex HRM Platform (Qiagen, Hilden, Germany) was used to perform the measurements.

### Bisulfite Conversion

DNA (500 ng) was treated with bisulfite using EZ DNA Methylation-Lightning Kit (Zymo Research). For MS-HRM and qMSP experiments, the concentration of BS converted DNA was measured by NanoDrop™ One/OneC Microvolume UV-Vis Spectrophotometer (Thermo Fisher Scientific, Waltham, MA, USA) and then adjusted to 10 ng·μl^− 1^.

### Primer Design

**Fig. 3:**
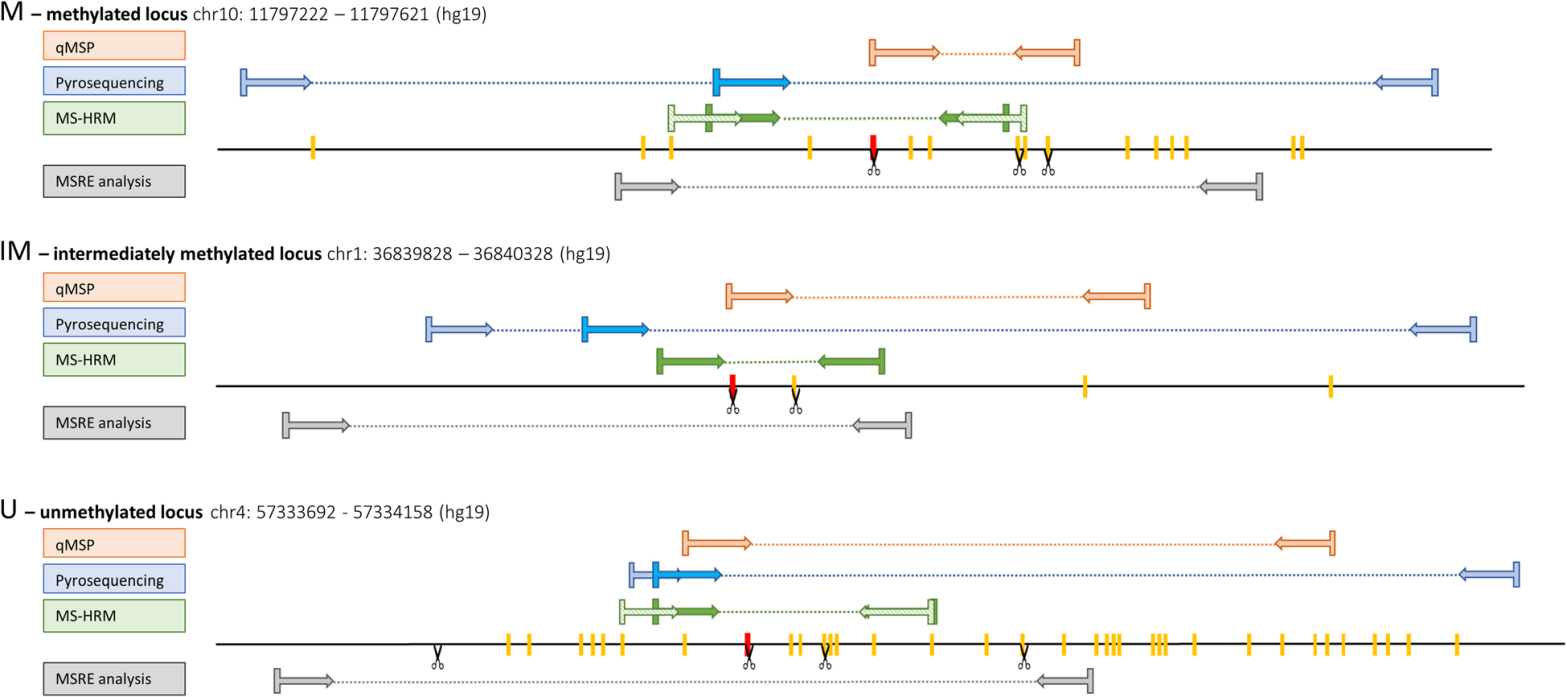
Positions of primer pairs, CpGs and restriction sites within studied regions. CpGs are shown as red and yellow bars on a line representing the DNA sequence. The red CpG is the one originally chosen from Infinium MethylationEPIC BeadChip. The scissors indicate sites that are cut by MSREs. The lighter blue primers were used for initial pyrosequencing PCR and the darker blue primers represent the sequencing primers. The patterned light green HRM primers were designed with a CpG on its 5’end (M/U Wojdacz primers)

For MSRE analysis, online software Primer3Plus (http://www.bioinformatics.nl/cgi-bin/primer3plus/primer3plus.cgi) was used. For methods that require BS converted DNA, we used Methyl Primer Express Software v1.0 (Thermo Fisher Scientific). For primers’ sequences and characteristics see Table 1. Positions of all primer pairs within the studied regions are shown in Fig. 3.

### Pyrosequencing

BS converted DNA (10–20 ng) was first amplified using HotStar HiFidelity Polymerase Kit (Qiagen) with final 2.5 mM MgCl_2_ concentration. To increase primers’ specificity and for easy gel loading, we added CoralLoad Concentrate from PyroMark PCR kit (Qiagen) to the final concentration of 1x. The final concentration of forward and universal biotinylated primer was 0.2 μM. The final concentration of reverse tailed primer was 0.04 μM. We used recommended PCR reaction conditions for PyroMark PCR with 48 PCR cycles and T_ann_ according to Table 1. Amplicon quality (1 μl of PCR reaction) was checked using 2% agarose gel electrophoresis. Pyrosequencing was performed on PyroMark Q24 instrument (Qiagen). Pyrosequencing protocol (User Manual 01/2016) was optimized by adding 2 μl of sepharose-coated Streptavidin beads (step 5.3.3.2) and by prolonging the agitation step to 20 min (step 5.3.3.6).

### MS-HRM Analysis

We prepared 100, 75, 50, 25, 10 and 0% methylated standards by mixing BS converted DNA methylated and unmethylated standards. 15 ng of BS converted samples and standards were processed using EpiTect HRM PCR Kit (Qiagen). Reaction conditions were set according to manufacturer’s protocol. The amount of reagents was adjusted to 20 μl final volume. The final concentration of primers was 0.375 μM. The experiment was performed on Rotor-Gene Q 2plex HRM Platform (Qiagen). For the HRM analysis, the ramping was set from 67.1 to 82.2 °C, rising by 0.1 °C/2 s. Raw data were processed using web-based tool uAnalyze [41]. In the software, we performed baseline normalization and calculated the difference curves for all standards and samples using the 0% methylated standard as a reference curve. Calibration curves were then plotted in Microsoft Excel according to Tse et al. [27] using peak heights and AUC of the standards’ processed data. From the calibration curves, the methylation percentage of analyzed samples was calculated.

### qMSP

Quantitative PCR with subsequent melting curve analysis was performed with 10–15 ng of BS converted DNA. Reaction mix (20 μl) was prepared using QuantiTect SYBR® Green PCR Kit (Qiagen). The final concentration of primers was 0.5 μM. We kept recommended cycling conditions with 40 cycles and T_ann_ according to Table 1. In one run, all samples together with methylated and unmethylated DNA standards were amplified with methylated MSP, unmethylated MSP and HRM primers. For each primer set, the amplification efficiency was calculated according to Dorak et al. [42]. We performed qPCR with four dilutions of BS converted DNA of one sample and plotted decadic logarithm of the dilutions against acquired C_t_s. The efficiency was then calculated from the slope of the calibration curve as follows: $E=\left[{10}^{\left(-\frac{1}{slope}\right)}-1\ \right]\bullet 100$. All measurements were done using StepOnePlus Real-Time PCR System (Thermo Fisher Scientific). Methylation levels were calculated using all three approaches reviewed in Husseiny et al. [36].

## Additional files

Additional file 1: Correlation coefficients for AUC-based MS-HRM calibration curves and counted methylation levels. (DOCX 14 kb)

Additional file 2: qMSP methylation results calculated using the relative expression ration. (DOCX 12 kb)

## Data Availability

The datasets supporting the conclusions of this article are included within the article (and its additional files).

## References

[1] Schubeler D (2015). Function and information content of DNA methylation. Nature.

[2] Jin Z, Liu Y (2018). DNA methylation in human diseases. Genes & Diseases.

[3] Levenson VV (2010). DNA methylation as a universal biomarker. Expert Rev Mol Diagn.

[4] Heyn H, Esteller M (2012). DNA methylation profiling in the clinic: applications and challenges. Nat Rev Genet.

[5] Pidsley R, Zotenko E, Peters TJ, Lawrence MG, Risbridger GP, Molloy P (2016). Critical evaluation of the Illumina MethylationEPIC BeadChip microarray for whole-genome DNA methylation profiling. Genome Biol.

[6] Lövkvist C, Dodd IB, Sneppen K, Haerter JO (2016). DNA methylation in human epigenomes depends on local topology of CpG sites. Nucleic Acids Res.

[7] Cedar H, Solage A, Glaser G, Razin A (1979). Direct detection of methylated cytosine in DNA by use of the restriction enzyme MspI. Nucleic Acids Res.

[8] Fraga MF, Esteller M (2002). DNA Methylation: A Profile of Methods and Applications. BioTechniques.

[9] Singer-Sam J, LeBon JM, Tanguay RL, Riggs AD (1990). A quantitative Hpall-PCR assay to measure methylation of DNA from a small number of cells. Nucleic Acids Res.

[10] Itoi E, Kokubun S, Hashimoto K, Roach HI (2007). Improved Quantification of DNA Methylation Using Methylation-Sensitive Restriction Enzymes and Real-Time PCR. Epigenetics.

[11] Kurdyukov S, Bullock M (2016). DNA Methylation Analysis: Choosing the Right Method. Biology.

[12] Untergasser A, Nijveen H, Rao X, Bisseling T, Geurts R, Leunissen JAM (2007). Primer3Plus, an enhanced web interface to Primer3. Nucleic Acids Res.

[13] Ye J, Coulouris G, Zaretskaya I, Cutcutache I, Rozen S, Madden TL (2012). Primer-BLAST: A tool to design target-specific primers for polymerase chain reaction. BMC bioinformatics.

[14] Hayatsu H, Wataya Y, Kazushige K (1970). Addition of sodium bisulfite to uracil and to cytosine. J Am Chem Soc.

[15] Sant KE, Nahar MS, Dolinoy DC. DNA methylation screening and analysis. Methods Mol Biol (Clifton, NJ). 2012;886:385–406. 10.1007/978-1-61779-867-2_24 PMC3592359 22669678

[16] Hernández HG, Tse MY, Pang SC, Arboleda H, Forero DA (2013). Optimizing methodologies for PCR-based DNA methylation analysis. BioTechniques.

[17] Frommer M, Mcdonald LE, Millar DS, Collis CM, Watt F, Grigg GW (1992). A genomic sequencing protocol that yields a positive display of 5-methylcytosine residues in individual DNA strands.

[18] Reed K, Poulin ML, Yan L, Parissenti AM (2010). Comparison of bisulfite sequencing PCR with pyrosequencing for measuring differences in DNA methylation. Anal Biochem.

[19] Tost J, El abdalaoui H, Glynne Gut I (2006). Serial pyrosequencing for quantitative DNA methylation analysis. BioTechniques.

[20] Delaney C, Garg SK, Yung R (2015). Analysis of DNA methylation by pyrosequencing. Methods Mol Biol.

[21] Tost J, Gut IG (2007). DNA methylation analysis by pyrosequencing. Nat Protoc.

[22] Li L.-C., Dahiya R. (2002). MethPrimer: designing primers for methylation PCRs. Bioinformatics.

[23] Arányi T, Váradi A, Simon I, Tusnády GE (2006). The BiSearch web server. BMC bioinformatics.

[24] King CR, Scott-Horton T, Marsh S (2007). Pyrosequencing. Pyrosequencing protocols Totowa.

[25] Guo D, Milewicz DM, Marsh S (2007). Universal primer applications for pyrosequencing. Pyrosequencing protocols Totowa.

[26] Wojdacz TK, Dobrovic A (2007). Methylation-sensitive high resolution melting (MS-HRM): a new approach for sensitive and high-throughput assessment of methylation. Nucleic Acids Res.

[27] Tse MY, Ashbury JE, Zwingerman N, King WD, Taylor SA, Pang SC (2011). A refined, rapid and reproducible high resolution melt (HRM)-based method suitable for quantification of global LINE-1 repetitive element methylation. BMC Res Notes.

[28] Smith E, Jones ME, Drew PA (2009). Quantitation of DNA methylation by melt curve analysis. BMC Cancer.

[29] Malentacchi F, Forni G, Vinci S, Orlando C (2009). Quantitative evaluation of DNA methylation by optimization of a differential-high resolution melt analysis protocol. Nucleic Acids Res.

[30] Wojdacz TK, Borgbo T, Hansen LL (2009). Primer design versus PCR bias in methylation independent PCR amplifications. Epigenetics.

[31] Wojdacz TK, Lotte HL (2006). Reversal of PCR bias for improved sensitivity of the DNA methylation melting curve assay. BioTechniques.

[32] Hansen LL, Wojdacz TK, Dobrovic A (2008). Methylation-sensitive high-resolution melting. Nat Protoc.

[33] Wojdacz TK, Hansen LL, Dobrovic A (2008). A new approach to primer design for the control of PCR bias in methylation studies. BMC Res Notes.

[34] Herman JG, Graff JR, Myöhänen S, Nelkin BD, Baylin SB (1996). Methylation-Specific PCR: A Novel PCR Assay for Methylation Status of CpG Islands. Proc Natl Acad Sci U S A.

[35] Derks S, Lentjes MH, Hellebrekers DM, de Bruïne AP, Herman JG, van Engeland M (2004). Methylation-specific PCR unraveled. Cellular oncology : the official journal of the International Society for Cellular Oncology.

[36] Husseiny MI, Kuroda A, Kaye AN, Nair I, Kandeel F, Ferreri K (2012). Development of a Quantitative Methylation-Specific Polymerase Chain Reaction Method for Monitoring Beta Cell Death in Type 1 Diabetes. PLoS One.

[37] Akirav EM, Lebastchi J, Galvan EM, Henegariu O, Akirav M, Ablamunits V (2011). Detection of β cell death in diabetes using differentially methylated circulating DNA. Proc Natl Acad Sci U S A.

[38] Eads CA, Danenberg KD, Kawakami K, Saltz LB, Blake C, Shibata D (2000). MethyLight: a high-throughput assay to measure DNA methylation. Nucleic Acids Res.

[39] Pfaffl MW (2001). A new mathematical model for relative quantification in real-time RT-PCR. Nucleic Acids Res.

[40] Šestáková Šárka, Krejčík Zdeněk, Folta Adam, Cerovská Ela, Šálek Cyril, Merkerová Michaela Dostálová, Pecherková Pavla, Ráčil Zdeněk, Mayer Jiří, Cetkovský Petr, Remešová Hana (2019). DNA methylation and hydroxymethylation patterns in acute myeloid leukemia patients with mutations in DNMT3A and IDH1/2 and their combinations. Cancer Biomarkers.

[41] Dwight Z, Palais R, Wittwer C (2012). uAnalyze: web-based high-resolution DNA melting analysis with comparison to thermodynamic predictions. IEEE/ACM Trans Comput Biol Bioinform.

[42] Pfaffl MW, Dorak MT (2007). Real-time qPCR amplification efficiency. Real-time PCR.

