# Supplementary material for: DNA Methylation Validation Methods: a Coherent Review with Practical Comparison
Source: Biol Proced Online. 2019 Oct 1;21:19. doi: 10.1186/s12575-019-0107-z (PMC6771119; doi:10.1186/s12575-019-0107-z)
Supplement: Supplementary file 1 — Correlation coefficients for AUC-based MS-HRM calibration curves and counted methylation levels. (DOCX 14 kb) [file 12575_2019_107_MOESM1_ESM.docx]

Correlation coefficients for AUC-based MS-HRM calibration curves and counted methylation levels

| Locus name | Primer set | R^2^ | Methylation [%]  (n = 10) | ± SD |
| --- | --- | --- | --- | --- |
| M | M HRM | 0.957 | 95.43 | 5.85 |
| M | M HRM Wojdacz | 0.846 | 148.58 | 8.68 |
| IM | IM HRM | 0.930 | 16.73 | 3.72 |
| U | U HRM | 0.852 | 1.95 | 0.69 |
| U | U HRM Wojdacz | 0.952 | 4.88 | 7.82 |

M - methylated locus, IM – intermediately methylated locus, U – unmethylated locus, R^2^ – square of the correlation coefficient, SD – standard deviation
