# Supplementary material for: DNA Methylation Validation Methods: a Coherent Review with Practical Comparison
Source: Biol Proced Online. 2019 Oct 1;21:19. doi: 10.1186/s12575-019-0107-z (PMC6771119; doi:10.1186/s12575-019-0107-z)
Supplement: Supplementary file 2 — qMSP methylation results calculated using the relative expression ration. (DOCX 12 kb) [file 12575_2019_107_MOESM2_ESM.docx]

qMSP methylation results calculated using the relative expression ration

| Locus and used primer set | Relative expression ratio | |
| --- | --- | --- |
|  | Average (n=10) | ± SD |
| M Met | 204.46 | 256.01 |
| IM Met | 1.92E-09 | 3.63E-09 |
| IM Unm | 0.02 | 0.02 |
| U Unm | 8.77 | 13.27 |

M - methylated locus, IM – intermediately methylated locus, U – unmethylated locus, Met – primers for methylated DNA sequence, Unm – primers for unmethylated DNA sequence, SD – standard deviation
